# Changes in the Protein Profile of Saliva from People with Obesity Treated with Bariatric Surgery and Physical Exercise

**DOI:** 10.3390/ijms26125622

**Published:** 2025-06-12

**Authors:** Margalida Monserrat-Mesquida, Maria Perez-Jimenez, Cristina Bouzas, Silvia García, Cláudia Mendes, Manuel Carvalho, Jorge Bravo, Sandra Martins, Armando Raimundo, Josep A. Tur, Elsa Lamy

**Affiliations:** 1 Physiopathology of Obesity and Nutrition (CIBEROBN), Instituto de Salud Carlos III, 28029 Madrid, Spain; margalida.monserrat@uib.es (M.M.-M.);; 2 Health Research Institute of Balearic Islands (IdISBa), 07120 Palma, Spain; 3 Research Group on Community Nutrition and Oxidative Stress, University of the Balearic Islands-IUNICS, IDISBA & CIBEROBN, Guillem Colom Bldg, Campus, 07122 Palma, Spain; 4 Mediterranean Institute for Agriculture Environment and Development (MED), Universidade de Évora, 7006-554 Évora, Portugal; maria.jimenez@uevora.pt (M.P.-J.);; 5 Unidade Local Saúde Alentejo Central—Hospital Espírito Santo de Évora, EPE, 7000-811 Évora, Portugal; 6 Centro Responsabilidade Integrada de Cirurgia da Obesidade e Metabólica (CRI.COM), 7000-811 Évora, Portugal; 7 Comprehensive Health Research Centre (CHRC), Universidade de Évora, 7002-554 Évora, Portugal; 8 Departamento de Desporto e Saúde, Escola de Saúde e Desenvolvimento Humano, Universidade de Évora, 7000-671 Évora, Portugal; 9 Universidade Lusófona’s Research Center for Biosciences & Health Technologie (CBIOS), 1749-024 Lisboa, Portugal; 10 Research Center in Sports Sciences, Health and Human Development( CIDESD), 4475-690 Maia, Portugal; 11 Faculty of Social Sciences and Technology, Universidade Europeia, 1749-016 Lisboa, Portugal

**Keywords:** saliva, protein profile, obesity, bariatric surgery, physical exercise

## Abstract

Saliva was used as non-invasive alternative to blood for diagnosing pathophysiological conditions. This study aimed to assess changes in protein profile in people with obesity after bariatric surgery and to assess the impact of exercise on these changes. The saliva proteome was determined from two-dimensional gels of twenty adults (ten people with normal weight and ten people with obesity). The effects of bariatric surgery and exercise were assessed. A decrease in body weight, body mass index, and waist-to-height ratio was observed after bariatric surgery. Low levels of carbonic anhydrase VI (CA-VI), short palate, lung, and nasal epithelium clone 2 (SPLUNC2), and haptoglobin were observed. One month after bariatric surgery, spots of haptoglobin and SPLUNC2 increased, although one CA-VI spot decreased. Zn-alpha-2 glycoprotein, immunoglobulin chains, and actin-related protein-3, which are high in people with obesity, decreased 1 month after bariatric surgery. Five months after bariatric surgery, the most significant change was the amylase decrease. The exercise-induced changes in salivary proteins increased SPLUNC, CA-VI, type S cystatins, actin cytoplasmic 1, and zinc alpha-2 glycoprotein levels and decrease Ig kappa chain C region and Rab GDP dissociation inhibitor beta. It can be concluded that the salivary proteins change between people with normal weight vs. patients with obesity, as well as after bariatric surgery and exercise programmes. Salivary proteins may be useful biomarkers in non-invasive samples for monitoring and assessing the impact of interventions on people with obesity.

## 1. Introduction

Obesity has become a major worldwide health problem for adults, as well as for children and adolescents, due to its exponentially growing prevalence in the last decades. In 2022, around 2.5 billion adults (43%) had overweight in the world, and 890 million of those (16%) presented obesity. Thus, one in eight people in the world showed obesity in 2022 [1]. Obesity is linked to a substantial decrease in life expectancy [2]. The increasing rates of obesity account for the heightened attention towards bariatric surgery. In contrast to non-surgical methods, bariatric surgery offers more significant and lasting benefits in terms of weight loss, a reduction in obesity-related complications, overall mortality rates, and quality of life [3]. Bariatric surgery causes some alterations, especially changes in various tissues, including the intestine, liver, pancreas, adipose tissue, and skeletal muscle, which contribute to overall improvements in insulin resistance, insulin secretion, and glucose metabolism independent of insulin, both with and without weight loss. Some patients may develop post-bariatric hypoglycemia months or even years after surgery, likely due to the significant reduction in glucose levels following the procedure [4].

Saliva was initially utilized as a non-invasive alternative to blood for diagnosing various pathophysiological conditions (e.g., some types of cancer, oral diseases, COVID-19, etc.), enhancing the understanding of these conditions [5]. Saliva has also been the subject of some studies on obesity, with individuals with obesity showing variations in this fluid compared to individuals with normal weight [6,7]. For example, a previous study observed that people with obesity showed lower salivary lipolysis compared to people with normal weight but had higher levels of proteolysis and carbonic anhydrase VI [6]. Changes in salivation after bariatric surgery have also been suggested by other authors who observed hypo-salivation in a considerable proportion (40%) of individuals submitted to bariatric surgery, together with changes in proteins such chromogranin A [7]. Saliva can be particularly valuable in obesity, since this has the potential to be a source of biomarkers [8,9,10,11].

Another reason saliva is particularly valuable for studying obesity is its involvement in the perception and ingestion of food in the mouth [5]. It was observed that more sensitivity to bitterness in children was related to higher BMI percentiles [12], and the salivary proteome (e.g., cystatins) is linked to bitter and sweet sensitivity in children, with this association varying based on BMI [12]. More recently, it was observed that people with obesity showed lower sensitivity to sweet and umami tastes, which was negatively correlated with the total protein content in their saliva [13]. Concerning bariatric surgery, several studies reported changes in the perception and preferences of food taste in individuals submitted to this procedure [14]. An increase in taste sensitivity to sweet and fatty stimuli and a lower acceptance of them post-surgery were also observed [14].

With this in mind, the variations in the salivary proteome observed in individuals with obesity submitted to bariatric surgery, namely the decreases in salivary amylase levels [5], could be related to the reported changes in the taste perception of these patients, which deserves to be further explored.

Exercise is advised post-bariatric surgery to minimize weight regain [15]. Previous studies have observed that exercise, including combined regimens and interventions starting after bariatric surgery, was effective in reducing body weight, waist circumference, and BMI. However, exercise after bariatric surgery does not appear to significantly enhance body composition [15]. Moreover, exercise training after bariatric surgery has beneficial effects because it enhances physical fitness, contributes to modest additional weight and fat loss, and may help prevent bone loss and weight regain [16].

The aim of this study was to understand the changes in the saliva protein profile in people with obesity treated with bariatric surgery and to assess the effect of physical exercise on them. This aim was divided into three specific objectives: to compare the saliva protein profile of people with normal weight vs. people with obesity; to assess the short-term alterations in the protein profile caused by bariatric surgery; and to assess the effect of a physical exercise intervention on the saliva protein profile.

## 2. Results

### 2.1. Anthropometric Parameters

Table 1 shows the anthropometric characteristics of participants stratified by different groups. Significant differences were observed in weight, BMI, waist circumference, and WHtR when comparing these measures before and after bariatric surgery, as well as between obese and normal-weight participants. After the bariatric surgery, the participants showed lower values in weight, BMI, waist circumference, and WHtR than before surgery, especially after four months of the intervention programme, but higher values than participants with normal weight (NW). Participants with obesity (OB) had a higher age than participants with NW. Participants from the exercise group were taller than those from the control group after 5 months post-surgery. Correlations between salivary protein variations and anthropometric changes in patients with obesity subjected to bariatric surgery are shown in Supplementary Table S1.

### 2.2. Differences in Salivary Proteome Between Individuals with Obesity vs. Individuals with Normal Weight

**Figure 1:**
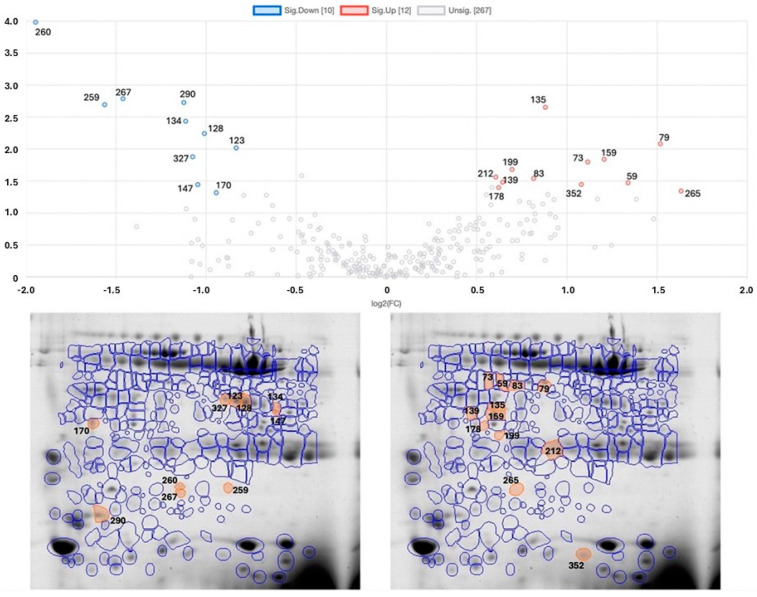
Volcano plot and representative 2D gel images comparing the salivary protein profiles of participants with obesity before bariatric surgery (OB-BS) and normal-weight participants (NW). The gel images represent one selected sample, chosen based on optimal spot resolution and considered by the software as the reference gel. Spots circled were the ones considered for analysis. The orange-marked and numbered spots are the ones showing statistically significant differences (fold change > 1.5; *p* < 0.05) between OB-BS and NW: on the left, spots that were decreased in OB-BS compared to NW; on the right, spots that were increased in OB-BS compared to NW. Molecular weight (MW) markers are shown on the vertical axis, and isoelectric point (pI) values are shown on the horizontal axis. The numbered spots correspond to identified proteins listed in Table 2.

Individuals with obesity showed changes in their saliva protein profile compared to individuals with normal weight. Thirteen protein spots were increased in the first group, whereas ten were present in lower amounts (Figure 1).

Among the spots for which salivary proteins were previously identified, proteins such as carbonic anhydrase VI (CA-VI) (four spots), prolactin-induced protein (PIP) (one spot), haptoglobin (two spots), and short palate, lung, and nasal epithelium clone (SPLUNC) (one spot) were decreased in participants with obesity, whereas Ig kappa chain C (two spots) and zinc-alpha-2 glycoprotein (three spots) were increased in participants with obesity compared to individuals with normal weight. Table 2 presents the results for these spots, which were able to be identified based on previous studies [17,18,19,20,21].

**Table 2:** Variations among groups/treatments in the salivary proteins identified for 2DE spots.

Spots	OB-BS vs. NW	OB-1M-BS	OB-5M-BS-Control	OB-5M-BS-Exercise	Protein ID	Ref
28			↓		Amylase	[18]
37			↓		Alpha-1 anti-trypsin	[18]
83	↑	↓			Actin-related protein 3	[19]
86				↑	Rab GDP dissociation inhibitor beta	[18]
96				↑	Actin cytoplasmic 1	[18,20]
110				↑	Zinc-alpha-2-glycoprotein	[18,20]
123	↓	↓			Carbonic-anhydrase VI	[18,20]
128	↓				Carbonic-anhydrase VI	[18,20]
134	↓				Carbonic anhydrase VI	[18]
135	↑				Zinc-alpha-2-glycoprotein	[17]
139	↑				Zinc-alpha-2-glycoprotein	[19]
159	↑				Zinc-alpha-2-glycoprotein	[17]
170	↓			↑	SPLUNC	[18]
177		↑			SPLUNC	[18]
212	↑			↓	Ig kappa chain C region	[18]
225	↑				Ig kappa chain C region	[18]
239				↓	Rab GDP dissociation inhibitor beta	[18]
259	↓				Haptoglobin	[18]
260	↓	↑			Haptoglobin	[18]
290	↓				PIP	[18]
310				↑	Cystatin SA	[21]
318			↓		Amylase (native)	[18,20]
321			↓		Amylase (native)	[18]
323			↓		Amylase (native)	[18]
327	↓			↑	Carbonic anhydrase VI	[18,20]
326		↓			Immunoglobulin J chain	[19]
333		↓			Zinc-alpha 2 glycoprotein	[17]

Abbreviations: BS, bariatric surgery; NW, participants with normal weight; OB-BS, participants with obesity before bariatric surgery; OB-1M-BS, obesity—1 month post-surgery; OB-5M-BS Control, obesity—5 months post-surgery (control group); OB-5M-BS Exercise, obesity—5 months post-surgery with exercise; ref., references; SPLUNC, nasal epithelium clone.

### 2.3. Effect of Bariatric Surgery on Saliva Proteome

**Figure 2:**
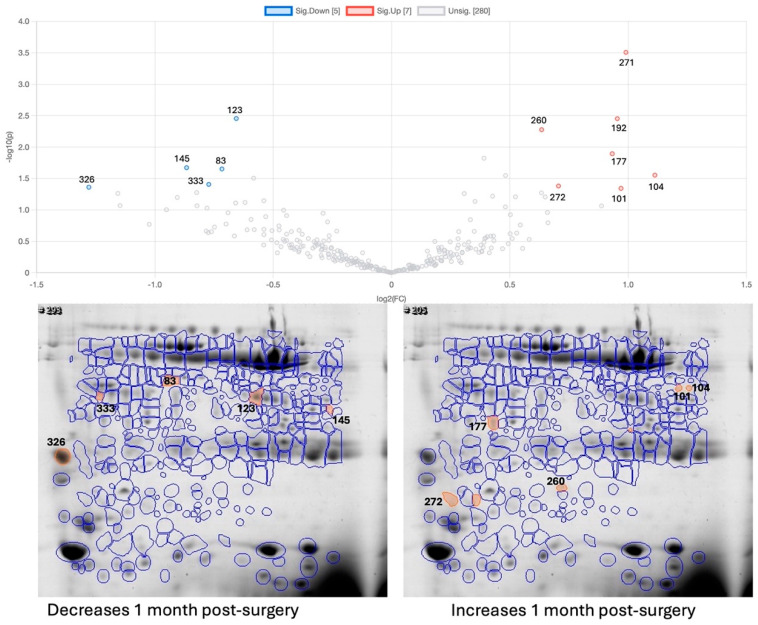
Vulcano plot and gel images for changes one month after bariatric surgery (OB-1M-BS) compared to the period before bariatric surgery (OB-BS); the orange-marked and numbered spots are the ones showing statistically significant differences: on the left are significant decreases and on the right are significant increases; MW—molecular mass marker; pI—isoelectric point.

One month after bariatric surgery, changes were observed for 12 protein spots (Figure 2). The spots of haptoglobin and SPLUNC increased in the participants post-surgery, compared to the period before surgery, and one spot of zinc-alpha 2 glycoprotein and one of immunoglobulin J chain decreased after bariatric surgery. Bariatric surgery also had a decreasing effect on one actin-related protein and one CA-VI spot. This latter spot corresponded to one of the spots that was already expressed in lower levels in participants with obesity compared to participants with normal weight (spot 123, Figure 1 and Table 1).

**Figure 3:**
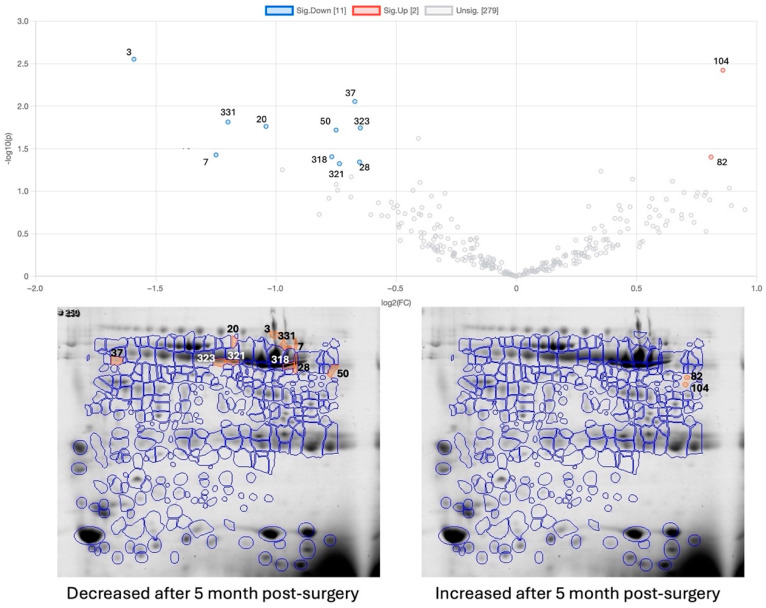
Vulcano plot and gel images for changes 5 months after bariatric surgery (OB-5M-BS) compared to the period before bariatric surgery (OB-BS); the orange-marked and numbered spots are the ones showing statistically significant differences: on the left are significant decreases and on the right are significant increases; MW—molecular mass marker; pI—isoelectric point.

Five months after bariatric surgery (and considering all the individuals, independent of having, or not having, an exercise intervention), one spot of alpha-1 anti-trypsin and four spots of amylase (three of them being the native form of amylase) were decreased compared to in the period before surgery (Table 2 and Figure 3).

Associations between the salivary proteins changed with bariatric surgery, and improvements in anthropometric parameters were observed for some protein spots. Spots 128 and 327 were negatively correlated with the percentage of total weight loss and positively correlated with the variation in BMI 1 month after bariatric surgery. In other words, these protein spot levels decreased more in the individuals with a higher percentage of total weight loss (and with a higher decrease in BMI). The correlations between changes in weight (BMI) and these salivary spots (identified as carbonic anhydrase VI) were observed mainly 1 month after bariatric surgery, suggesting that the salivary changes directly related to improvements in anthropometric parameters were more pronounced during the first stage post-surgery. Increases in the levels of spots 86 (identified as Rab GDP dissociation inhibitor beta) and 290 (identified as PIP) 5 months post-surgery tended to be lower in the individuals with higher decreases in waist circumference.

### 2.4. Effect of Exercise Intervention on the Saliva Proteome of Bariatric-Surgery-Submitted Participants

**Figure 4:**
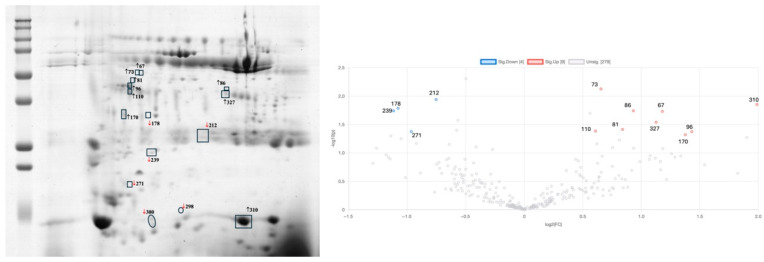
Variations induced by exercise after bariatric surgery (OB-1M-BS vs. OB-5M-BS): (**left**) gel image with statistically significant different spots signaled, where circles signal the 2 spots changed in OB-5M-BS-Control and squares signal the spots changed in OB-5M-BS-Exercise; (**right**) volcano plot showing differences between OB-1M-BS-Exercise and OB-5M-BS-Exercise; MW—molecular mass marker; pI—isoelectric point).

The effect of an exercise programme on the salivary proteome was assessed by comparing the saliva collected at the beginning of the programme (1 month after surgery) and after 4 months of the intervention. Almost no variations in saliva were seen in the control group, with only two spots (with apparent molecular masses near 15 kDa) decreasing. However, in the group submitted to the exercise programme, increases in SPLUNC, CA-VI, type S cystatins, actin cytoplasmic 1, and zinc alpha-2 glycoprotein and decreases in Ig kappa chain C region and Rab GDP dissociation inhibitor beta were observed compared to the beginning of the intervention (Table 2 and Figure 4).

## 3. Discussion

The current results are in accordance with previous studies that evidenced a body weight loss after bariatric surgery [22]. Like these results, a BMI reduction was reported in participants undergoing bariatric surgery [23]. WHtR was a predictor of risk of cardiovascular disease and mortality among obesity and type 2 diabetes patients [24]. In the current study, a lower level of WHtR was observed after 4 months of intervention, indicating a reduction in the risk of cardiovascular disease among the participants after bariatric surgery.

Saliva is a fluid showing variations with different pathological and physiological conditions, and the current results reinforce that obesity is associated with differences in the salivary protein profile, in line with previous studies [5,20].

Among the proteins present at different levels between people with normal weight and individuals with obesity before being submitted to surgery, CA-VI was one for which variations with obesity were previously observed [5,6,20]. In a previous study, a positive correlation between the levels of this protein and BMI was observed for women with morbid obesity [5]. Although none of the CA-VI protein spots showed this association in the current study, when the data was analysed for the period before surgery, the decreases in CA-VI were associated with decreases in total weight loss and BMI, with higher decreases in individuals that lost more weight and that had higher decreases in BMI. The current results are in line with a previously reported study concerning the effect of bariatric surgery in decreasing the relative amounts of this salivary protein [5]. CA-VI is the only secreted isoenzyme of carbonic anhydrases, being produced by serous acinar cells in the parotid and submandibular glands [25]. This protein was linked to taste sensitivity [25], with decreased levels in gustatory and olfactory disfunction, and, in this context, better knowledge about its association with obesity may be interesting. Different proteoforms of CA-VI have been identified for saliva, although little is known about the different functions of these [26].

Prolactin-inducible protein (PIP) was lower in the women with obesity compared to the women with normal weight. Although not much information exists about the levels of this protein in individuals with obesity, some studies linked this protein to sweet and bitter taste sensitivity, namely by showing higher levels in individuals with low sensitivity to bitterness and high sensitivity to sweetness [20,27]. This is a secretory protein present in exocrine secretions, like saliva, and its expression is induced by the peptide hormone prolactin and by androgens, being downregulated by oestrogen [28]. Although the exact biological role of this protein is not totally understood, data suggests that PIP can have immunomodulatory functions, participating in innate immunity and protection of the host against microbial infections [29].

Haptoglobin and SPLUNC were present at lower levels in participants with obesity, but their levels increased one month after bariatric surgery. SPLUNC, also named Parotid Secretory Protein (PSP), has been considered as a host defence protein, due to its lipid-binding capacity [30]. Haptoglobin is a glycoprotein involved in the acute-phase response to inflammation, and some studies reported higher levels of this protein in the blood of individuals with obesity [31]. However, it seems that this protein may be more linked to hyperinsulinemia than BMI itself [31]. It has been observed that a deficiency of haptoglobin is related to obesity-associated insulin resistance and hepatosteatosis [32]. The reason why the levels of this protein are higher in saliva from individuals with obesity and decrease with bariatric surgery, in opposition to what would be expected from previously reported results of blood, needs to be further clarified.

Zinc-alpha 2 glycoprotein, which was present at higher levels in the saliva of the participants with obesity in the current study, has also been previously observed at higher levels in saliva from women with obesity compared to women after bariatric surgery [5]. Higher serum levels of this protein were found for individuals with obesity in other studies [33], and it has been reported that it plays an important role in the pathophysiology of obesity, being associated with metabolic syndrome [34]. Furthermore, it seems that zinc-alpha 2 glycoprotein may play a role in regulating lipid metabolism in adipose tissue and is linked to insulin resistance [35]. The observation in the current study of the decrease in the levels of one spot of this protein after bariatric surgery suggests improvements in metabolism even 1 month after surgery.

The positive effects of bariatric surgery can also be inferred from the decreased levels of chains of immunoglobins, in opposition to the period before surgery, where saliva from individuals with obesity showed higher levels of these chains than saliva from people with normal weight. In fact, before surgery, two spots containing Ig kappa chain C region were present at higher levels in saliva from individuals with obesity, and 1 month after bariatric surgery, it was the spot identified as Immunoglobulin J chain that decreased in individuals after surgery. The J chain is essential for the formation of dimeric IgA, which is required for the secretion of this protein in the mucosa. The presence of immunoglobulins, including IgA, was positively correlated with BMI and fat % in previous studies [36].

Beyond the effects of surgery alone, our findings also provide insights into the impact of exercise in the post-surgical period. The salivary protein profile of individuals who participated in the post-surgery exercise intervention revealed further distinct changes compared to those who had only undergone surgery. Specifically, proteins such as SPLUNC, CA-VI, cystatin SA, actin cytoplasmic 1, and zinc-alpha-2 glycoprotein increased in the OB-5M-BS-Exercise group, while immunoglobulin kappa chain C region and Rab GDP dissociation inhibitor beta decreased. These changes were not observed in the OB-5M-BS-Control group, suggesting that physical exercise may independently contribute to the modulation of salivary proteins associated with immune regulation and metabolic processes [16,37]. Notably, some of these proteins (e.g., cystatins and CA-VI) have been previously associated with taste perception and satiety regulation, which may indicate an additional mechanism through which exercise supports weight maintenance after surgery [20,26]. These findings highlight the potential of salivary proteins as biomarkers to differentiate between the biological effects of bariatric surgery alone and those enhanced by structured physical activity.

Additionally, regarding the individuals with obesity, 5 months after surgery, one of the main changes in the saliva was in amylase. This decrease was not statistically significant 1 month after surgery. The effect of decreases in salivary amylase of bariatric surgery has already been reported in previous studies [5]. Salivary alpha amylase is a protein secreted by the acinar cells of serous salivary glands. This enzyme, which participates in the oral digestion of starch, was also linked to food oral perception. Sweet taste sensitivity was negatively correlated with the levels of this protein in previous studies [27].

The levels of this protein in saliva have been seen to decrease in animal models of non-obesity-induced hyperleptinemia [38], suggesting this decrease was correlated with an increase in the satiety hormone. Considering that bariatric surgery improves leptin sensitivity [39], higher leptin (and other satiety hormone) signalling may be linked to the decreases in the levels of this salivary protein. Since amylase participates in starch digestion, it is possible to hypothesize that some months after bariatric surgery, the oral digestion of starch decreases, which may have consequences for the metabolism and weight management of these individuals. This is an interesting point to explore in future works. It is also important not to exclude a dietary explanation. Because the dietary habits of these patients changed, and since diet is a source of changes in the saliva proteome [40], it is important to investigate at what level the different changes in the salivary proteome are linked to the changes in food intake.

This study was designed as a pilot exploratory trial, with a limited sample size. Therefore, the primary aim was to detect preliminary trends and identify potential salivary protein biomarkers associated with bariatric surgery and exercise. The findings presented here lay the groundwork for future studies with larger and more representative cohorts, which will be essential to validate and generalize these observations.

### Strengths and Limitations

The principal strength of this study lies not only in the comparison between individuals with obesity and normal-weight participants, but also in the longitudinal analysis of the same individuals with obesity at multiple time points—prior to bariatric surgery and at two follow-up stages post-surgery. In addition, the study design allowed for the evaluation of the effects of a structured physical exercise intervention on a subset of participants.

However, several limitations must be acknowledged. First, the sample size was relatively small, which limits the statistical power and generalizability of the findings. Another important limitation is the age difference between the normal-weight (NW) group and the individuals with obesity before bariatric surgery (OB-BS). Due to the constraints of clinical recruitment and the specific eligibility criteria for bariatric surgery, it was not feasible to fully age-match the groups. Given that salivary protein expression may be influenced by age-related physiological changes, this discrepancy could act as a confounding factor in the interpretation of proteomic differences. Future studies with larger, age-matched cohorts will be necessary to determine whether the observed variations are attributable to obesity and related interventions, rather than age alone.

Additionally, this study included only female participants to reduce sex-related variability in salivary proteome profiles and metabolic responses. While this approach improved internal consistency, it limits the generalizability of the results to males. Future research should include both sexes to validate these findings and explore possible sex-specific patterns in salivary biomarkers.

A further methodological limitation is that protein identification was not conducted de novo via mass spectrometry. Instead, spot identities were inferred based on comparisons with previously published 2D-GE salivary proteomes. Although this approach allowed for a practical and cost-effective exploratory analysis, it lacks the specificity and confirmatory strength of mass-spectrometry-based methods. Future studies should include MS validation to strengthen protein identification accuracy.

Finally, this study did not include detailed assessments of dietary intake or comorbidity status. While all participants received standard post-surgical nutritional counselling, no food diaries or dietary records were collected, and comorbid conditions such as diabetes or hypertension were not systematically evaluated. As both diet and comorbidities can influence salivary protein composition, their omission may have introduced additional variability. Future research should incorporate comprehensive dietary and clinical assessments to better isolate the effects of obesity and its treatment on the salivary proteome.

## 4. Material and Method

### 4.1. Study Design

Ten women been 18 and 65 years of age residing in Évora (Portugal) were included in this study. Participants indicated whether they met the following inclusion criteria: (1) registration for bariatric surgery at the Espirito Santo Hospital in Évora; (2) body mass index (BMI) between 35 and 50 kg/m^2^; (3) no contraindication to exercise; (4) agreed to participate in the programme. Ten normal-weight women participants in the same age range were also included in this study as healthy controls without obesity. So, this study was carried out with a total of 20 women. Only female participants were included in this study in order to minimize biological variability related to sex-specific differences in salivary proteome composition and metabolic responses to obesity and bariatric surgery. This approach aimed to increase internal consistency in this exploratory phase of research. All participants with obesity included in this study underwent Roux-en-Y gastric bypass surgery, which was the standard bariatric procedure performed at the Espirito Santo Hospital in Évora during the recruitment period. This surgical approach was uniformly applied to ensure consistency in the type of metabolic intervention received by the participants.

All participants received standard post-surgical nutritional counselling according to hospital protocol. However, no formal dietary intake records were collected during the study period, and no structured screening for comorbidities such as diabetes, hypertension, or dyslipidaemia was conducted. These factors were not included in the analysis.

The participants with obesity in this study were divided into two groups: one receiving the regular healthy recommendations that are provided to any individual that undergoes bariatric surgery (control group), and one receiving a physical exercise programme (exercise group). Whereas the saliva of normal-weight individuals was only collected at one point, the participants with obesity underwent three evaluations: the first was before the surgery (baseline), the second was one month post-surgery before starting the exercise programme, and the third was five months post-surgery, four months after the exercise programme. In each of these three evaluations, saliva was collected.

The study protocols adhered to the ethical standards outlined in the Declaration of Helsinki and received approval from the Ethics Committee of University of Evora and the Espiritu Santo Hospital in Évora (HESE_CE_1917/21; 14 October 2021). All participants were informed about the purpose and implications of this study and provided written consent to participate. This study was registered in Clinicals Trials.gov, ref. NCT05289219 [17].

### 4.2. Anthropometric Parameters

Professional observers conducted anthropometric measurements to minimize variations between different observers. Height and weight were measured using a wall-mounted stadiometer and high-quality electronic scales, respectively. Height was recorded with a mobile anthropometer (Seca 214, SECA Deutschland, Hamburg, Germany) to the nearest millimetre, ensuring the patient’s head was in the Frankfort horizontal plane position. Body weight was assessed with a Segmental Body Composition Analyzer (Tanita MC 780-P MA, Hoogoorddreef 56E 1101 BE Amsterdam, The Netherlands) following the manufacturer’s guidelines. Subjects were weighed barefoot and in light clothing, with 0.6 kg subtracted to account for their clothing. These measurements were used to calculate the body mass index (BMI) by dividing the weight in kilograms by the square of height in metres. Waist circumference (WC) was measured twice at the midpoint between the last rib and the iliac crest using an anthropometric tape. The waist-to-height ratio (WHtR), an indicator of cardiovascular risk, was calculated by dividing waist circumference (cm) by height (cm).

### 4.3. Whole-Saliva Collection

Prior to conducting taste tests, unstimulated whole saliva was obtained using the drooling technique. Following this, each participant rinsed their mouth with water and was then instructed to expel all saliva produced over a period of 4 min into a tube kept on ice. The collected saliva was subsequently frozen at −20°C. After that, samples were thawed on ice and then subjected to centrifugation at 13,000× *g* for 15 min at 4 °C to eliminate mucinous and insoluble material.

### 4.4. Protein Total Determination

The Bradford method was used for determining the total protein concentration of the supernatant fraction of all saliva samples. Bovine serum albumin (BSA) was used as the standard from a stock solution of 2000 µg/mL. Saliva samples and standards were placed in 96-well microplates, and then mixed with Bradford reagent. Finally, the absorbance was measured at 600 nm using a microplate reader (Glomax, Promega, Madison, WI, USA). A standard curve of known concentrations was used to calculate the total protein concentration.

### 4.5. Two-Dimensional Electrophoresis

Two-dimensional gel electrophoresis (2-DE) analysis was conducted for the saliva of ten normal-weight participants and ten individuals with obesity undergoing bariatric surgery; of these ten patients with obesity, five of them were in the control group (receiving regular healthy recommendations), while the other five were part of the intervention group (physical exercise programme). In addition, individual gel analysis was carried out for each of the three evaluations of the patients undergoing bariatric surgery. The protein load was 150 μg for each individual gel.

Saliva samples were freeze-dried overnight and then mixed with rehydration buffer composed of 7M urea, 2M thiourea, 4% (*w*/*v*) CHAPS [3-(3-cholamidopropyldimethylammoniun)-1 propanesulfonate], 2% (*v/v*) IPG buffer, 60 mM dithiothreitol (DTT), and bromophenol blue 0.002% (*w*/*v*) to a final volume of 125 μL. Then, samples were incubated at room temperature for one hour. After that, samples were centrifugated at 10 000 rpm for 5 min at room temperature. The supernatant from each sample was applied to the slots of the strip holder of the Multiphor II system (GE healthcare, Buc, Yvelines, France), and commercial gel strips (pH gradient 3–10 NL 7 cm (IPG strips, GE healthcare)) were placed in contact with the sample, remaining in passive rehydration overnight at room temperature and covered with mineral oil (dry strip cover fluid, GE healthcare). After rehydration, the strips were placed in the Multiphor II system for isoelectric focusing of the proteins (first dimension). Focusing occurred at a constant temperature of 18 °C according to the following programme: step 1—rise to 100 V (0:01 h), step 2—300 V (1:00 h), step 3—rise to 3500 V (4:00 h), step 4—3500 V (3:00 h).

Following focusing, proteins in the IPG strips were reduced by soaking in 1% (*w*/*v*) DTT;75mMTris–HCl, pH 8.8; 6 M urea; 29,3% (*v/v*) glycerol; and 2% (*w*/*v*) SDS. Then, they were alkylated with 4% (*w*/*v*) iodoacetamide; 75 mM Tris–HCl, pH 8.8; 6 M urea; 29,3% (*v/v*) glycerol; and 2% (*w*/*v*) SDS. The equilibrated strips were then horizontally applied on the top of a 14% SDS-PAGE gel (1 × 160 × 160 mm), and proteins were separated vertically at 18 °C, using a Protean II xi cell (Bio-Rad) at a constant voltage of 150 V until the end of the run. Molecular masses were determined in accordance with molecular mass standards (Bio-Rad Precision Plus Protein Dual Colour 161–0394; Hercules CA, USA) run with protein samples. Gels were fixed for 1 h in 40% methanol/10% acetic acid, followed by staining for 2 h with Coomassie Brilliant Blue (CBB) G-250. Gel images were acquired using a scanning Molecular Dynamics densitometer with internal calibration and LabScan software (GE Healthcare), and images were analysed using SameSpots (TotalLab) software. A total of 43 gels were analysed, and the volume of each spot was normalised using relative spot volumes (% vol.). Gels were aligned, considering as reference a 2DE profile with good spot resolution and where most of the spots that could be considered for analysis were observed. Manual alignment was followed by automatic alignment, considering the manual indications provided.

The decision to use two-dimensional gel electrophoresis (2D-GE) in this study was based on its ability to resolve intact proteins and proteoforms with high reproducibility, particularly in complex biological fluids such as saliva. While mass-spectrometry-based methods (e.g., LC-MS/MS) offer depth of proteome coverage and sensitivity, 2D-GE allows for the direct visualization of post-translational modifications (PTM). This capability is particularly relevant for investigating functional protein changes associated with obesity, metabolic alterations, and taste perception, where PTMs and proteoform diversity may play critical roles. Moreover, considering the relatively small sample size of this study and its exploratory nature, 2D-GE was a cost-effective and informative choice for highlighting major protein expression changes across groups.

### 4.6. Exercise Programme

The exercise was part of a progressive combined training programme that included strength and aerobic training in a single session. Information on frequency, intensity, time, type, volume, and progression (FITT-VP) was incorporated in the exercise prescription [40].

For 16 weeks, patients allocated to the IG group participated in a combined exercise training programme. A 5 min targeted warm-up, phase 1 resistance training (weeks 1–4), phase 2 hypertrophy training (weeks 5–10), phase 3 strength training (weeks 11–16), and a 10 min flexibility cool-down (myofascial release, mobility, static and dynamic stretching) were all included in each exercise session.

Three personal trainers with backgrounds in sports sciences evaluated the participants’ level of physical fitness and recommended and supervised training regimens. Every training session was conducted in a fitness centre, three times a week on non-consecutive days, from seven to ten in the morning.

### 4.7. Statistics

The Statistical Package for Social Sciences (SPSS v.29 for Windows, IBM Software Group, Chicago, IL, USA) was used to describe the characteristic parameters for participants. Results are presented as means ± standard deviation (SD), considering a *p*-value < 0.05 as statistically significant. One-way analysis of variance (ANOVA) was used to check the significance of the data. A Bonferroni post hoc test was conducted when significant differences were identified between the groups.

For proteomics data, statistical analysis and data visualization were performed using Metaboanalyst 6.0 software. The 292 protein spots considered for analysis (after 2-DE gel image analysis), were included. Since data presented no normal distribution, log2 transformation was applied. To identify the protein spots with significant differences between groups, a Student’s *t*-test was used. For comparing samples from individuals with obesity vs. those with normal weight, independent samples were considered, whereas for comparisons between each of the periods after bariatric surgery and the period before (only for participants with obesity), paired data was assumed. In each case, Volcano plots were displayed, where the resulting −log10 (*p*-value) values were displayed versus the spots’ (percentage volume) fold change. Only the spots with fold changes > 1.5 and *p*-values < 0.05 were signalled. For the spots showing differences between NW participants and OB participants due to bariatric surgery, Pearson correlation was performed to assess the existence of an association between variation in anthropometry and variation in salivary parameters.

## 5. Conclusions

The salivary proteins are different between people with normal weight vs. patients with obesity, as well as after bariatric surgery and exercise programmes. This innovative study allowed us to observe that some of these changes depended on the time that had passed since surgery, as well as on lifestyle. Taking part in an exercise programme in the post-surgery period appeared to have effects on the level of salivary proteins that were potentially associated with taste perception and metabolism, suggesting further potential effects of the success of the obesity treatment intervention. Salivary proteins may be helpful biomarkers as non-invasive samples for monitoring and evaluating the effects of interventions on people with obesity.

## Figures and Tables

**Table 1:** Characteristics of participants according to different groups.

	NW (n = 10)	OB (n = 10) (Before BS)	OB-1M-BS (n = 10)	OB-5M-BS-Control (n = 5)	OB-5M-BS Exercise (n = 5)	ANOVA
	Mean ± SD	Mean ± SD	Mean ± SD	Mean ± SD	Mean ± SD	*p*-Value
Age (years)	34.3 ± 9.6	49.1 ± 11.1 ^a^	49.1 ± 11.1 ^a^	54.0 ± 10.7 ^a^	44.2 ± 10.1	0.007
Height (cm)	164.6 ± 6.1	158.9 ± 8.5	158.9 ± 8.5	151.8 ± 5.7 ^a^	166.0 ± 1.6 d	0.013
Weight (cm)	62.6 ± 9.9	102.7 ± 15.1 ^a^	90.9 ± 13.5 ^a^	66.2 ± 8.2 ^b,c^	79.0 ± 9.6 ^b^	<0.001
BMI (kg/m^2^)	23.2 ± 3.9	40.5 ± 3.4 ^a^	35.9 ± 3.8 ^a^	28.7 ± 2.6 ^b,c^	28.7 ± 3.5 ^b,c^	<0.001
Waist (cm)	77.6 ± 6.8	120.9 ± 11.6 ^a^	109.2 ± 11.8 ^a^	91.2 ± 13.6 b	93.2 ± 11.4 b	<0.001
WHtR	0.471 ± 0.044	0.764 ± 0.103 ^a^	0.690 ± 0.094 ^a^	0.602 ± 0.095 ^b^	0.562 ± 0.071 ^b^	<0.001

Results are expressed as mean ± SD. Abbreviations: BMI, body mass index; BS, bariatric surgery; NW, participants with normal weight; OB, participants with obesity; OB-1M-BS, obesity—1 month post-surgery; OB-5M-BS Control, obesity—5 months post-surgery (without exercise); OB-5M-BS Exercise, obesity—5 months post-surgery with exercise; WHtR, waist-to-height ratio; SD: standard deviation. Statistical analysis by one-way ANOVA and Bonferroni test. ^a^ Differences regarding normal weight. ^b^ Differences regarding participants with obesity before BS. ^c^ Differences 1 month after BS. ^d^ Differences 5 months post-surgery (control).

## Data Availability

There are restrictions on the availability of data for this trial, due to the signed consent agreements around data sharing, which only allow access to external researchers for studies following the project purposes. Researchers wishing to access the trial data used in this study can make a request to pep.tur@uib.es.

## Supplementary Materials

The following supporting information can be downloaded at https://www.mdpi.com/article/10.3390/ijms26125622/s1.
