# Supplementary material for: Changes in the Protein Profile of Saliva from People with Obesity Treated with Bariatric Surgery and Physical Exercise
Source: Int J Mol Sci. 2025 Jun 12;26(12):5622. doi: 10.3390/ijms26125622 (PMC12193444; doi:10.3390/ijms26125622)
Supplement: Supplementary file 1 [file ijms-26-05622-s001.zip › ijms-3642822-Supplementary Material.pdf]

Supplementary Material

Table S1. Correlations between salivary protein variations and anthropometric changes in patients with obesity subjected to bariatric surgery.

	% Total weight loss (1st month)	Delta BMI (BMI 1M – BMI initial)	Delta waist (BMI 1M – BMI initial)
128 (OB-1M-BS – 128 OB-BS)	R= -.642 (P=.045)	R=.634 (P=.049)	
327 (OB-1M-BS – 128 OB-BS)	R= -.704 (P=.023)	R=.655 (P=.040)	
239 (OB-1M-BS – 128 OB-BS)			R=-.711 (P=.021)
	% Total weight loss (5th month)	Delta BMI (BMI 5M – BMI initial)	Delta waist (BMI 5M – BMI initial)
86 (OB-5M-BS – 128 OB-BS)			R=.750 (P=.013)
290 (OB-5M-BS – 128 OB-BS)			R=.691 (P=.027)

Abbreviations: BMI body mass index; BS, Bariatric Surgery; OB, participants with obesity; OB-1M-BS, Obesity – 1 Month Post-Surgery; OB-5M-BS, Obesity- 5 Months post-surgery. Pearson correlation was performed to access the existence of association between variation in anthropometry and variation in salivary parameters.
